# Inflammation-induced hepcidin-25 is associated with the development of anemia in septic patients: an observational study

**DOI:** 10.1186/cc9408

**Published:** 2011-01-10

**Authors:** Lucas T van Eijk, Joyce JC Kroot, Mirjam Tromp, Johannes G van der Hoeven, Dorine W Swinkels, Peter Pickkers

**Affiliations:** 1 Department of Intensive Care Medicine, Radboud University Nijmegen Medical Centre, Nijmegen, Geert Grooteplein-Zuid 10, P.O. Box 9201, 6500 HB Nijmegen, The Netherlands; 2 Department of Clinical Chemistry, Radboud University Nijmegen Medical Centre, Nijmegen, Geert Grooteplein-Zuid 10, P.O. Box 9201, 6500 HB Nijmegen, The Netherlands; 3 Department of Internal Medicine, Radboud University Nijmegen Medical Centre, Nijmegen, Geert Grooteplein-Zuid 10, P.O. Box 9201, 6500 HB Nijmegen, The Netherlands; 4 Nijmegen Institute for Infection, Inflammation, and Immunity (N4i), Radboud University Nijmegen Medical Centre, Nijmegen, Geert Grooteplein-Zuid 10, P.O. Box 9201, 6500 HB Nijmegen, The Netherlands

## Abstract

**Introduction:**

Anemia is a frequently encountered problem during inflammation. Hepcidin is an interleukin-6 (IL-6)-induced key modulator of inflammation-associated anemia. Human sepsis is a prototypical inflammatory syndrome, often complicated by the development of anemia. However, the association between inflammation, hepcidin release and anemia has not been demonstrated in this group of patients. Therefore, we explored the association between hepcidin and sepsis-associated anemia.

**Methods:**

92 consecutive patients were enrolled after presentation on the emergency ward of a university hospital with sepsis, indicated by the presence of a proven or suspected infection and ≥ 2 extended systemic inflammatory response syndrome (SIRS) criteria. Blood was drawn at day 1, 2 and 3 after admission for the measurement of IL-6 and hepcidin-25. IL-6 levels were correlated with hepcidin concentrations. Hemoglobin levels and data of blood transfusions during 14 days after hospitalisation were retrieved and the rate of hemoglobin decrease was correlated to hepcidin levels.

**Results:**

53 men and 39 women with a mean age of 53.3 ± 1.8 yrs were included. Hepcidin levels were highest at admission (median[IQR]): 17.9[10.1 to 28.4]nmol/l and decreased to normal levels in most patients within 3 days (9.5[3.4 to 17.9]nmol/l). Hepcidin levels increased with the number of extended SIRS criteria (P = 0.0005). Highest IL-6 levels were measured at admission (125.0[46.3 to 330.0]pg/ml) and log-transformed IL-6 levels significantly correlated with hepcidin levels at admission (r = 0.28, P = 0.015), day 2 (r = 0.51, P < 0.0001) and day 3 (r = 0.46, P < 0.0001). Twelve patients received one or more blood transfusions during the first 2 weeks of admission, not related to active bleeding. These patients had borderline significant higher hepcidin level at admission compared to non-transfused patients (26.9[17.2 to 53.9] vs 17.9[9.9 to 28.8]nmol/l, P = 0.052). IL-6 concentrations did not differ between both groups. Correlation analyses showed significant associations between hepcidin levels on day 2 and 3 and the rate of decrease in hemoglobin (Spearman's r ranging from -0.32, P = 0.03 to -0.37, P = 0.016, respectively).

**Conclusions:**

These data suggest that hepcidin-25 may be an important modulator of anemia in septic patients with systemic inflammation.

## Introduction

Inflammation-associated anemia represents an important and highly prevalent clinical problem. In 2000, Krause et al. described a peptide that was later called 'hepcidin' based on its hepatic expression and antimicrobial activity [1,2]. This β-defensin-like peptide was found to be a principle regulator of systemic iron homeostasis. In concordance with this dual function, its expression is modulated by systemic iron requirements and inflammatory stimuli, as it is induced by cytokines such as IL-6 [3]. Its role in the development of anemia was first suggested in 2001 [4]. Since then it has been demonstrated that hepcidin is a central modulator of inflammation-associated anemia, not only by controlling the expression of ferroportin on intestinal cells and macrophages [5], but also via a direct inhibitory effect of hepcidin on erythropoiesis [6]. In humans, increased concentrations of hepcidin were detected in patients with chronic infections and severe inflammatory diseases [7]. The association of increased concentrations of hepcidin with anemia has been determined in patients suffering from chronic inflammation [8], chronic kidney disease [9], and cancer [10]. In addition, acute systemic inflammation evoked by experimental endotoxemia in humans resulted in an increase in hepcidin release, associated with a decrease in serum iron [11]. Nevertheless, the association between the innate immune response, hepcidin release and consequent decrease in hemoglobin (Hb) has not been established in patients with an acute systemic inflammation.

Human sepsis is a prototypical acute inflammatory syndrome frequently complicated by the development of anemia. As the incidence of sepsis is high [12], determination of this putative pathway of the development of anemia is of clinical importance. Therefore, we explored the correlation between IL-6 and hepcidin, and the subsequent rate of Hb decrease and number of blood transfusions received in septic patients.

## Materials and methods

### Subjects and sampling

This is an explorative observational study in which data of the subjects were retrieved from a prospectively aggregated database of patients with sepsis. Following Dutch law, the local Institutional Review Board of Arnhem-Nijmegen indicated that no formal approval was required for this study. Patients were informed, but no written consent was necessary. Ninety-two consecutive septic patients were enrolled after presentation on the emergency ward and subsequent hospital admission. Sepsis was defined by the presence of two or more extended criteria for systemic inflammation (body temperature >38.3 or < 36°C, acutely altered mental status, shivering, heart rate >90 bpm, systolic blood pressure < 90 mmHg or mean arterial pressure < 65 mmHg, respiratory rate >20 breaths/min, hyperglycemia in absence of diabetes) and a proven or suspected source of infection [13]. The total number of extended systemic inflammatory response syndrome (SIRS) scores were calculated. Patients were given usual care according to the guidelines of the Surviving Sepsis Campaign [14]. Blood was drawn at day one, two and three of admission for the measurement of IL-6 and hepcidin-25. Hb measurements were not taken as part of a protocol, but Hb levels that were determined as part of standard hospital care during the first 14 days after hospital admission were retrieved and used for further analysis, Hemoblobin levels were checked regularly, but not every day in every patient. Also the accompanying indices of mean corpuscular volume (MCV), mean cell Hb (MCH), and red cell distribution width (RDW) were analyzed. Blood transfusions during hospital stay were recorded. We hypothesized that the effect of sustained elevated levels of hepcidin could be first seen in the Hb level after a period of 7 to 14 days. This was based on the assumption that erythrocytes circulate for approximately 120 days. If erythropoiesis would be abrogated by hypoferremia due to an increased hepcidin level, it would therefore take approximately 12 days to reduce the Hb levels by 10%. A decrease of 10% was considered a clinically relevant and reliably detectable difference. However, due to a possible direct inhibitory effect of hepcidin on erythropoiesis, and a reduced erythrocyte half-life during inflammation, a detectable reduction of Hb from day seven onwards was anticipated.

### Laboratory measurements

IL-6 levels were measured on an Immulite 2500 (Siemens, Breda, The Netherlands), based on a solid-phase, enzyme-labelled, chemiluminescent sequential immunometric method. Serum hepcidin-25 measurements were performed by a combination of weak cation exchange chromatography and time-of-flight mass spectrometry (TOF-MS), using a Microflex LT matrix-enhanced laser desorption/ionisation TOF-MS platform (Bruker Daltonics, Bremen, Germany). An internal standard (synthetic hepcidin-24; Peptide International Inc., Louisville, KT, USA) was used for quantification [15,16].

### Calculations and statistical analysis

Log-transformed IL-6 concentrations were correlated with hepcidin-25 concentrations using Pearson's correlation coefficient. Hepcidin-25 was correlated with the rate of decrease of Hb between day 1 and 14, using Spearman's correlation coefficient. The rate of decrease of Hb was calculated per patient by linear regression using all available Hb measurements. If Hb levels were not measured at days 7 to 14, or if patients received a blood transfusion during their stay, they were excluded from this analysis. Hepcidin levels at admission (prior to any transfusion) of patients who received a blood transfusion during the first 14 days of hospitalization were compared with patients who did not.

To test whether the presence of comorbidity affected the rate of Hb decrease, we divided different forms of comorbidity into eight categories (chronic kidney disease, hematologic, malignancy, pulmonary, rheumatic/autoimmune, cardiologic, urologic, and other) and performed a step-wise multi-variate analysis in which hepcidin levels and the eight categories of comorbidity were added to the model. If a comorbidity was found to significantly attribute to the prediction of Hb decrease, it was left in the model, but otherwise discarded. Data are expressed as mean ± standard error of the mean or median (25^th ^to 75^th ^percentile) depending on their distribution. Correlations were expressed as Spearman's correlation coefficient, except for the correlations between hepcidin and log-transformed IL-6 concentrations that were expressed as Pearson's r.

Paired observations over time were tested with Wilcoxon matched-pairs test and unpaired observations with a Mann-Whitney test.

## Results

### Demographic data

Demographic data of the subjects are displayed in Table 1. Two patients died during hospitalization. Blood culture results are presented in Table 2. Twenty percent of the patients had a positive blood culture. This relatively low percentage is probably due to the fact that in most cases the general practitioner had already initiated antimicrobial therapy before admission to the hospital.

**Table 1:** Demographic data of the subjects

	Number (%)
Total	92 (100)
Male/female	53/39 (58/42)
Age (years)	53.3 ± 1.8
ICU admissions	3 (3)
Deaths	2 (2)
Median hospital length of stay (days)	6 (4-11)
Number of SIRS criteria present	2.5 ± 0.9
Number of patients transfused	12 (13)
Site of infection	
Lung	28 (30)
Abdomen	12 (13)
Urinary tract	24 (26)
Skin/soft tissue	4 (4)
Bone/joint	3 (3)
Blood	2 (2)
Cerebral	1 (1)
Other	3 (3)
Unknown	9 (10)
No infectious focus	6 (7)
Comorbidity	
None	36 (39)
Chronic kidney disease	13 (14)
Hematologic disease	7 (8)
Malignancy	8 (9)
Lung disease	6 (7)
Rheumatic / autoimmune disease	2 (2)
Cardial disease	1 (1)
Urological disease	5 (5)
Other	14 (15)

Data are expressed as absolute numbers and percentages of total, mean ± standard error of the mean or median (25^th ^to 75^th ^percentile). Multivariate analysis demonstrated that comorbidities were not independently associated with hemoglobin decrease. SIRS, systemic inflammatory response syndrome.

**Table 2:** Blood culture results

Organisms and culture sites	Number of patients
Organisms	
*Aeromonas *species	1
*Candida *species	2
*Citrobacter *species	1
*Corynebacterium jeikeium*	1
*Coxiella *species	4
*Enterobacter *species	1
*Enterococcus *species	5
*Escherichia coli*	10
*Haemophilus influenzae*	1
*Klebsiella *species	2
*Morganella *species	1
*Pseudomonas *species	3
*Salmonella *species	1
*Staphylococcus *species	2
*Streptococcus *species	3
Viral infection (positive serological test)	6
No pathogen cultured	50
Sites	
Blood	18
Urine	18
Other	4
Multiple organisms	2
Multiple sites	2

### IL-6 and hepcidin

**Figure 1:**
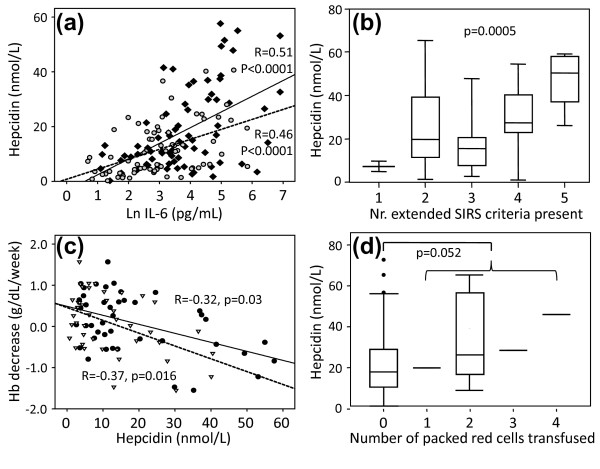
**Association between IL-6, hepcidin and hemoglobin decrease**. **(a) **Humoral relation between inflammation and hepcidin levels: Pearson's correlation between the natural logarithm (Ln) of IL-6 and hepcidin-25 on day 2 (black diamonds, uninterrupted line), and day 3 (grey dots, dashed line. The correlation on day 1 (r = 0.28, *P *= 0.015), was omitted for reasons of clarity. The median reference level of serum hepcidin-25 is 4.2 nM, range 0.5 to 13.9 nM [15]. **(b) **Clinical relation between inflammation and hepcidin levels: hepcidin-25 levels according to the number of extended systemic inflammatory response syndrome (SIRS) criteria at presentation at the emergency ward [13]. Differences were tested with Kruskal-Wallis. **(c) **Spearman's correlation between rate of hemoglobin (Hb) decrease and hepcidin-25 concentration on day 2 (black dots, uninterrupted line) and day 3 (open triangles, dashed line). The rate of decrease was only calculated in patients that did not receive a blood transfusion and of whom Hb was measured at least once between day 7 and 14 of hospital admission (*n* = 44). **(d) **Relation between hepcidin-25 levels at admission and the number of blood transfusions received during 14 days of follow up. Boxes represent median and interquartile range, whiskers represent 5^th ^and 95^th ^percentile. Difference between transfused and non-transfused patients was tested with a Mann Whitney test.

IL-6 was highest at admission (125.0 (46.3 to 330.0) pg/ml), and decreased on day two (37.2 (16.8 to 112.8) pg/ml) and day three (19.5 (7.4 to 55.7) pg/ml). A similar pattern was observed for hepcidin levels, being highest at admission (17.9 (10.1 to 28.4) nmol/l) and declining to 9.5 (3.4 to 17.9) nmol/l on day three, which is still increased compared with control values. Log-transformed IL-6 levels correlated significantly with hepcidin levels on admission, day two and day three, (Pearson's r = 0.28, *P *= 0.015; r = 0.512, *P*< 0.0001; r = 0.458, *P*< 0.0001, respectively; Figure 1a). Also, the number of extended SIRS criteria present correlated with hepcidin levels (Figure 1b).

### Hepcidin and hemoglobin

Hb was 12.0 (11.2 to 13.4) g/dl at admission and decreased to an average of 11.3 (10.3 to 12.8) g/dl at day 7 to 14 (*P *= 0.004) in patients who did not receive a blood transfusion. During hospitalization the Hb levels decreased at least 0.8 g/dl in 69 (86%) of 80 patients who did not receive a blood transfusion. There was no correlation between hepcidin levels and Hb levels at admission (r = 0.21, *P *= 0.07). Hepcidin levels on day one of admission did not correlate with the rate of decrease in Hb (r = -0.13, *P *= 0.39). Hepcidin on day two and day three significantly correlated with the rate of decrease of Hb (r = -0.32, *P *= 0.03 and r = -0.37, *P *= 0.016; Figure 1c).

Twelve patients received one or more blood transfusions during the first two weeks of admission, not related to active bleeding. These patients had borderline significant higher hepcidin level at admission (preceding any blood transfusion) compared with non-transfused patients (26.9 (17.2 to 53.9) vs 17.9 (9.9 to 28.8)nmol/l, *P *= 0.052; Figure 1d).

MCV slightly increased during hospital admission from 86.0 (84.0 to 90.0) to an average of 88.5 (85.7 to 92.6) fl at day 7 to 14 (*P *= 0.011). RDW increased from 13.9 (13.1 to 15.4) to an average of 15.9 (14.2 to 17.0)% (*P *= 0.002). MCH remained unchanged during 14 days of follow up (from 1.84 (1.75 to 1.90) fmol to an average of 1.82 (1.76 to 1.90) fmol at day 7 to 14 (*P *= 0.39)). None of the changes in red cell indices correlated with the hepcidin levels on days one to three.

## Discussion

In the present study, three novel findings emerged. This is the first study to show that: hepcidin-25 is increased during human sepsis; in septic patients the degree of inflammation, indicated by IL-6 levels and number of SIRS criteria present, is associated with the elevated concentrations of hepcidin; and persistently increased levels of hepcidin-25 at day two and day three after admission are associated with a decrease in Hb during hospitalization. Naturally, in patients who received a transfusion, the effect of hepcidin on Hb could not be determined and these patients were excluded from this part of the analysis. In a separate analysis, we showed that transfused patients showed a trend towards higher hepcidin levels than those who were not. These findings combined suggest that the observed association between elevated hepcidin and a decrease in Hb is likely to be an underestimation.

This study does not necessarily indicate a causal relation between elevated hepcidin and Hb decrease in septic patients. However, the causal relation of the induction of hepcidin by IL-6 and the development of anemia by sustaining elevated hepcidin levels has been shown by others in separate experiments [3,5,6,17]. This study is the first to address the combined measurement of hepcidin, IL-6 and Hb levels in patients and shows that hepcidin probably plays a role in sepsis-associated anemia. One may argue that the correlation between inflammation, hepcidin and anemia is an epiphenomenon, because more severely affected patients release higher levels of inflammatory parameters and also receive more fluids during their volume resuscitation, resulting in the more pronounced decrease in Hb. This appears not to be the case, because hepcidin concentrations at admission did not predict the decrease in Hb, in contrast to more prolonged elevations in hepcidin during the first three days. Moreover, the decrease in Hb was determined over 14 days, a period in which the effects of volume resuscitation should have diminished. Nevertheless, it is important that in addition to the physiological role of hepcidin, several other factors that lead to anemia during infection have been described, such as iatrogenic blood loss, inhibition of erythropoietin production [18], blunted erythropoietic response [18,19], and a decreased lifespan of erythrocytes, mediated by increased adherence to the vascular wall and phagocytosis by macrophages [20]. In addition, in these patients the presence of different comorbidities and the severity of the disease could have influenced the development of anemia. However, we were not able to express these variables in size and number, and therefore could not include these parameters as a continuous variable into a multivariate regression analysis. This may explain the relatively low correlation coefficients we found between hepcidin and Hb decrease.

There are two known ways that hepcidin can result in inflammation-associated anemia. First, hepcidin can abrograte erythroid colony formation in situations where erythropoietin concentrations are reduced, as is the case during sepsis [6]. Furthermore, inflammation leads to sequestration of iron in cells resulting in a blocked transport of iron to the bone marrow. Considering the fact that the lifespan of erythrocytes of approximately 120 days might be shortened due to inflammation and the fact that hepcidin suppresses erythropoiesis itself, we hypothesized that if hepcidin is upregulated by inflammation and thereby suppresses serum iron levels, a measurable effect on Hb level was expected from seven days onwards after the diagnosis of sepsis. Furthermore, we anticipated a swift decrease in inflammation in treated septic patients. For these reasons we determined IL-6 and hepcidin levels for three days and the rate of Hb decrease within 7 to 14 days.

We were not able to demonstrate a relation of Hb decrease with a change in MCV, MCH, or RDW. This does not invalidate the hypothesis that increased hepcidin attributes to the development of anemia in these patients. It was previously shown that erythrocyte progenitor cells carry iron transporter ferroportin1B on their cell membrane [21]. During inflammation systemically elevated hepcidin down-regulates ferroportin on these cells, thereby preventing a loss of intracellular iron and the microcytic anemia that is seen in iron-deficiency anemia. Therefore, inflammation-associated anemia is not typically microcytic [22]. Moreover, the observed effect of hepcidin on the development on anemia may have been mediated by a direct inhibitory effect on erythropoiesis, rather than by blocking iron transport to the bone marrow by sequestration.

Interestingly, hepcidin at day one did not predict the rate of Hb decrease. Probably persistently elevated hepcidin levels are necessary to exert a relevant effect on Hb concentrations. The association we found may be an underestimation, because the patients in this study were already anemic at the time of presentation to the emergency ward and likewise it is possible that before presentation hepcidin levels were even more pronounced. Nevertheless, although statistically significant, the observed association between hepcidin and Hb levels is modest, indicating that other previously mentioned factors that influence Hb are likely to play a role.

## Conclusions

Anemia during acute systemic inflammation evoked by sepsis is a frequently encountered clinical problem. Up to now, human data concerning the effect of hepcidin release on the development of anemia during sepsis were absent. Our study demonstrates that inflammation in septic patients is associated with increased hepcidin-25 concentrations. Moreover, the elevated hepcidin concentrations observed in early sepsis negatively correlated with Hb levels during the hospital stay of these patients. These human *in vivo *correlations suggest that hepcidin release is a modulator of anemia in septic patients with systemic inflammation.

## Key messages

• IL-6 concentrations and number of SIRS criteria present in septic patients are associated with increased hepcidin-25 concentrations.

• The increase in hepcidin concentrations observed in early sepsis correlates with the decrease in Hb levels during their hospital stay and patients with higher hepcidin concentrations tend to need more blood transfusions.

• The inflammation-hepcidin release-anemia pathway is present in patients with sepsis.

## Abbreviations

Hb: hemoglobin; IL-6: interleukin 6; MCH: mean cell hemoglobin; MCV: mean corpuscular volume; RDW: red cell distribution width; SIRS: systemic inflammatory response syndrome; TOF-MS: time-of-flight mass spectrometry.

## Competing interests

The authors declare that they have no competing interests.

## Authors' contributions

LTE participated in data collection, performed the statistical analysis and drafted the manuscript. JJCK performed hepcidin measurements and participated in drafting the manuscript. MT collected demographic and SIRS data, built the database and collected the blood samples. JGH revised the manuscript and participated in the design of the study. DWS revised the manuscript and participated in the design of the study and was responsible for hepcidin measurements. PP conceived of the study, participated in its design and coordination and helped to draft the manuscript.

## References

[B1] Krause A Neitz S Magert HJ Schulz A Forssmann WG Schulz-Knappe P Adermann K LEAP-1, a novel highly disulfide-bonded human peptide, exhibits antimicrobial activity FEBS Lett 2000 480 147 150 10.1016/S0014-5793(00)01920-7 11034317

[B2] Park CH Valore EV Waring AJ Ganz T Hepcidin, a urinary antimicrobial peptide synthesized in the liver J Biol Chem 2001 276 7806 7810 10.1074/jbc.M008922200 11113131

[B3] Nemeth E Rivera S Gabayan V Keller C Taudorf S Pedersen BK Ganz T IL-6 mediates hypoferremia of inflammation by inducing the synthesis of the iron regulatory hormone hepcidin J Clin Invest 2004 113 1271 1276 15124018 10.1172/JCI20945 PMC398432

[B4] Fleming RE Sly WS Hepcidin: a putative iron-regulatory hormone relevant to hereditary hemochromatosis and the anemia of chronic disease Proc Natl Acad Sci USA 2001 98 8160 8162 10.1073/pnas.161296298 11459944 PMC37412

[B5] Nemeth E Tuttle MS Powelson J Vaughn MB Donovan A Ward DM Ganz T Kaplan J Hepcidin regulates cellular iron efflux by binding to ferroportin and inducing its internalization Science 2004 306 2090 2093 10.1126/science.1104742 15514116

[B6] Dallalio G Law E Means RT Jr Hepcidin inhibits in vitro erythroid colony formation at reduced erythropoietin concentrations Blood 2006 107 2702 2704 10.1182/blood-2005-07-2854 16332970 PMC1895381

[B7] Cherian S Forbes DA Cook AG Sanfilippo FM Kemna EH Swinkels DW Burgner DP An insight into the relationships between hepcidin, anemia, infections and inflammatory cytokines in pediatric refugees: a cross-sectional study PLoS ONE 2008 3 e4030 10.1371/journal.pone.0004030 19107209 PMC2603326

[B8] Theurl I Aigner E Theurl M Nairz M Seifert M Schroll A Sonnweber T Eberwein L Witcher DR Murphy AT Wroblewski VJ Wurz E Datz C Weiss G Regulation of iron homeostasis in anemia of chronic disease and iron deficiency anemia: diagnostic and therapeutic implications Blood 2009 113 5277 5286 10.1182/blood-2008-12-195651 19293425

[B9] Ashby DR Gale DP Busbridge M Murphy KG Duncan ND Cairns TD Taube DH Bloom SR Tam FW Chapman RS Maxwell PH Choi P Plasma hepcidin levels are elevated but responsive to erythropoietin therapy in renal disease Kidney Int 2009 75 976 981 10.1038/ki.2009.21 19212416

[B10] Ukarma L Johannes H Beyer U Zaug M Osterwalder B Scherhag A Hepcidin as a predictor of response to epoetin therapy in anemic cancer patients Clin Chem 2009 55 1354 1360 10.1373/clinchem.2008.121285 19406916

[B11] Kemna E Pickkers P Nemeth E van der HH Swinkels D Time-course analysis of hepcidin, serum iron, and plasma cytokine levels in humans injected with LPS Blood 2005 106 1864 1866 10.1182/blood-2005-03-1159 15886319

[B12] Dombrovskiy VY Martin AA Sunderram J Paz HL Rapid increase in hospitalization and mortality rates for severe sepsis in the United States: a trend analysis from 1993 to 2003 Crit Care Med 2007 35 1244 1250 10.1097/01.CCM.0000261890.41311.E9 17414736

[B13] Levy MM Fink MP Marshall JC Abraham E Angus D Cook D Cohen J Opal SM Vincent JL Ramsay G 2001 SCCM/ESICM/ACCP/ATS/SIS International Sepsis Definitions Conference Crit Care Med 2003 31 1250 1256 10.1097/01.CCM.0000050454.01978.3B 12682500

[B14] Nguyen HB Rivers EP Abrahamian FM Moran GJ Abraham E Trzeciak S Huang DT Osborn T Stevens D Talan DA Severe sepsis and septic shock: review of the literature and emergency department management guidelines Ann Emerg Med 2006 48 28 54 16781920 10.1016/j.annemergmed.2006.02.015

[B15] Kroot JJ Hendriks JC Laarakkers CM Klaver SM Kemna EH Tjalsma H Swinkels DW (Pre)analytical imprecision, between-subject variability, and daily variations in serum and urine hepcidin: implications for clinical studies Anal Biochem 2009 389 124 129 10.1016/j.ab.2009.03.039 19341701

[B16] Swinkels DW Girelli D Laarakkers C Kroot J Campostrini N Kemna EH Tjalsma H Advances in quantitative hepcidin measurements by time-of-flight mass spectrometry PLoS ONE 2008 3 e2706 10.1371/journal.pone.0002706 18628991 PMC2442656

[B17] Lee P Peng H Gelbart T Wang L Beutler E Regulation of hepcidin transcription by interleukin-1 and interleukin-6 Proc Natl Acad Sci USA 2005 102 1906 1910 10.1073/pnas.0409808102 15684062 PMC548537

[B18] Jelkmann W Proinflammatory cytokines lowering erythropoietin production J Interferon Cytokine Res 1998 18 555 559 10.1089/jir.1998.18.555 9726435

[B19] Rogiers P Zhang H Leeman M Nagler J Neels H Melot C Vincent JL Erythropoietin response is blunted in critically ill patients Intensive Care Med 1997 23 159 162 10.1007/s001340050310 9069000

[B20] Kempe DS Akel A Lang PA Hermle T Biswas R Muresanu J Friedrich B Dreischer P Wolz C Schumacher U Peschel A Götz F Döring G Wieder T Gulbins E Lang F Suicidal erythrocyte death in sepsis J Mol Med 2007 85 273 281 10.1007/s00109-006-0123-8 17180345

[B21] Zhang DL Hughes RM Ollivierre-Wilson H Ghosh MC Rouault TA A ferroportin transcript that lacks an iron-responsive element enables duodenal and erythroid precursor cells to evade translational repression Cell Metab 2009 9 461 473 10.1016/j.cmet.2009.03.006 19416716 PMC2685206

[B22] Keel SB Abkowitz JL The microcytic red cell and the anemia of inflammation N Engl J Med 2009 361 1904 1906 10.1056/NEJMcibr0906391 19890136 PMC3741048

